# Metabolome Analysis of *Drosophila melanogaster* during Embryogenesis

**DOI:** 10.1371/journal.pone.0099519

**Published:** 2014-08-14

**Authors:** Phan Nguyen Thuy An, Masamitsu Yamaguchi, Takeshi Bamba, Eiichiro Fukusaki

**Affiliations:** 1 Department of Biotechnology, Graduate School of Engineering, Osaka University, Suita, Osaka, Japan; 2 Department of Applied Biology, Insect Biomedical Research Center, Kyoto Institute of Technology, Sakyo-ku, Kyoto, Japan; CINVESTAV-IPN, Mexico

## Abstract

The *Drosophila melanogaster* embryo has been widely utilized as a model for genetics and developmental biology due to its small size, short generation time, and large brood size. Information on embryonic metabolism during developmental progression is important for further understanding the mechanisms of *Drosophila* embryogenesis. Therefore, the aim of this study is to assess the changes in embryos’ metabolome that occur at different stages of the *Drosophila* embryonic development. Time course samples of *Drosophila* embryos were subjected to GC/MS-based metabolome analysis for profiling of low molecular weight hydrophilic metabolites, including sugars, amino acids, and organic acids. The results showed that the metabolic profiles of *Drosophila* embryo varied during the course of development and there was a strong correlation between the metabolome and different embryonic stages. Using the metabolome information, we were able to establish a prediction model for developmental stages of embryos starting from their high-resolution quantitative metabolite composition. Among the important metabolites revealed from our model, we suggest that different amino acids appear to play distinct roles in different developmental stages and an appropriate balance in trehalose-glucose ratio is crucial to supply the carbohydrate source for the development of *Drosophila* embryo.

## Introduction

Developmental biology is one of the most challenging fields for biologists since the mechanism governing the development of an organism from a single cell still remains unclear. As an important model organism [1], [2], *Drosophila* embryos have been commonly used to investigate the function of genes related to biological pathways occurring during its development such as cell proliferation, differentiation and apoptosis [3]. After fertilization, *Drosophila* embryo undergoes thirteen cycles of rapid, highly synchronized nuclear division to form a syncytium in the absence of cytokinesis. Following these nuclear division cycles, each nucleus at the cortex surface is simultaneously packaged into individual cells in a process known as cellularization. Afterwards, the single-layered cellular blastoderm is then rearranged during gastrulation to produce an embryo composed of three primordial tissue layers [4]. Although many genes related to developmental processes have been identified and the gene expression database for *Drosophila* embryo is now available online [5], it is still not clear how the gene products participate in various cellular processes. On the other hand, metabolites, the end products of various cellular processes in a living cell or living organism are particularly good indicators for an organism’s phenotype or physiology [5]. Thus, metabolomics, one of the latest “omics” technology concerned with the high throughput identification and quantification of metabolites, is indispensable in elucidating the mechanism underlying *Drosophila* embryogenesis.

In fact, several metabolomics studies have been conducted using *Drosophila* that focused on the effect of heat tolerance on third instar larvae [6], [7] and adult flies as well as [8]–[11] hypoxia tolerance [12], pheromones [13], oxidative stress [14], longevity [15] and obesity [16] in *Drosophila* larvae and adults. Furthermore, metabolomics using *Drosophila* as model organism has been applied for the study of *Listeria monocytogenes* infection [17] and drug efficacy test [18]. In these studies, several techniques have been applied for metabolic profiling of *Drosophila* larvae or adults, such as Liquid Chromatography Fourier Transform Mass [19], [20], Liquid Chromatography-tandem Mass Spectrometry with Liquid Chromatography-Multiple Reaction Monitoring [21] or ion pairing Liquid Chromatography/Mass Spectrometry [22]. However, since all of these studies were carried out in *Drosophila* larvae or adults, up to now the information on the metabolic profiling of *Drosophila* during embryogenesis is still unclear. In this study, we have succeeded in establishing the metabolic profiling of *Drosophila melanogaster* during embryogenesis by analyzing the low molecular weight metabolites with gas chromatography quadrupole mass spectrometry (GC-Q/MS). We also found that distinct metabolic profiling correlated with different stages of *Drosophila* embryogenesis. We constructed a Partial Least Square projection to the latent structure (PLS) model to predict the embryo stages and propose the important metabolites for the development of *Drosophila* embryo. To our knowledge, this is the first report of a robust and accurate regression model based on a high resolution quantitative metabolome analysis of *Drosophila* embryos.

## Materials and Methods

### Fly strain and embryo collection

Canton S, a wild type strain of *Drosophila melanogaster*, was reared on Instant *Drosophila* Medium (Wako, Japan). The collecting embryo step was done by using the method as described previously [23], [24]. After the virgin flies were collected, flies with different genders were kept separately for 3 days until they become mature enough for mating. Mating was subsequently done overnight in egg collecting cages on agar plates containing standard food (Dry yeast 50 g/L; Glucose 50 g/L; Agar 15 g/L) with freshly prepared yeast paste. The following day, the plates were exchanged every two hours and plates from the first two hours were discarded to clear the eggs laid by flies overnight. Then, embryos were incubated at 25°C in which samples were collected at different time points specifically 0–2 hrs, 2–4 hrs, 4–6 hrs, 6–8 hrs, 8–10 hrs, 10–12 hrs, 12–14 hrs, 14–16 hrs, 16–18 hrs, 18–20 hrs AEL (hours After Egg Laying). After collection, the samples were washed with NaCl-Triton (0.7% NaCl; 0.03% Triton X-100) five times and subsequently washed three times with H_2_O. Embryos were then frozen in liquid N_2_ immediately and freeze-dried overnight. Finally, all samples were stored at −30°C before extraction.

### Sample extraction and derivatization for GC-MS

The freeze-dried sample was crushed using a ball mill for 5 min at 20 Hz before extraction to increase the extraction efficiency. Afterwards, 5 mg of each sample was extracted with 1 mL extraction solvent, which consisted of methanol/water/chloroform (2.5∶1∶1). 60 µL ribitol (0.2 mg/mL) was added subsequently as internal standard. After centrifugation at 16,000×g for 3 min at 4°C, 900 µL of the supernatant was transferred to a 1.5 mL micro tube and mixed with 400 µL distilled water (Wako). After repeating centrifugation, 400 µL of the polar phase was transferred into a fresh 1.5 mL microfuge tube with a screw cap. Then, the solvent was removed using a centrifugal concentrator (VCe36S, Taitec Co., Tokyo, Japan) for 2 hours and sample was subsequently freeze-dried overnight.

Derivatization of the samples was done by oximation using methoxyamine hydrochloride (Sigma Aldrich, St. Louis, MO, USA) in pyridine (50 µL, 10 mg/mL) at 30°C for 90 min, followed by silylation using 25 *µ*L of N-methyl-N- (trimethylsilyl) trifluoroacetamide (MSTFA) (GL Sciences, Tokyo, Japan) at 37°C for 30 min. Three samples at the same time point were analyzed (n = 3) and each of them was collected independently from different set of parents at the same conditions.

### GC-MS analysis

Gas chromatography quadrupole mass spectrometry (GC-Q/MS) analysis was performed on GCMS- QP 2010 Ultra (Shimadzu) with a CP-SIL 8 CB low-bleed column (0.25 mm×30 m, 0.25 µm, Varian Inc., Palo Alto, CA, USA) and an AOC-20i/s autosampler (Shimadzu). Tuning and calibration of the mass spectrometer was done prior to analysis. One microliter of derivatized sample was injected in split mode, 25∶1 (v/v), with an injection temperature of 230°C. The carrier gas (He) flow was 1.12 mL/min with a linear velocity of 39 cm/s. The column temperature was held at 80°C for 2 min, increased by 15°C/min to 330°C, and then held for 6 min. The transfer line and ion source temperatures were 250 and 200°C, respectively. Ions were generated by electron ionization (EI) at 0.94 kV. Spectra were recorded at 10000 u/s (check value) over the mass range m/z 85−500. A standard alkane mixture (C8−C40) was injected at the beginning and end of the analysis for tentative identification.

### Data processing

The raw chromatographic data were converted into ANDI files (Analytical Data Interchange Protocol,*.cdf) using GC-MS Solution software package (Shimadzu). The data were imported to MetAlign software [25], [26] (Wageningen UR, The Netherlands, available for free at the website http://www.pri.wur.nl/UK/products/MetAlign/) for peak selection and alignment. The peak intensity of each compound was normalized based on the ribitol internal standard. AIoutput2 (version 1.29) was used as annotation software. The retention indices of all detected metabolites were calculated based on the standard alkane mixture and tentative identification of metabolites was done by comparing the retention indices with our in-house library [27] to aid the tentative identification of compounds. On the other hand, the retention time of each metabolite was used to compare with the NIST 2011 Library (NIST11/2011/EPA/NIH).

### Multivariate analysis

A heatmap of identified metabolites was established using the Multiexperiment Viewer Version 4.9 [28] (Dana-Farber Cancer Institute, Boston, MA, USA, available for free at the web site http://www.tm4.org/mev.html). The agglomerative hierarchical cluster analysis was utilized based on Pearson correlation with gene leaf optimization and complete linkage clustering.

PCA (Principal Component Analysis) and PLS (Partial Least Square projections to latent structures) were performed by utilizing SIMCA-P^+^ version 11 (Umetrics, Umea, Sweden). First, PCA was utilized to summarize, classify and discriminate the large amount of data acquired. Then, PLS was utilized for modeling relationships between the metabolome and *Drosophila* embryogenesis. Pareto scaling method was used and transformation was not performed.

### Identification of important metabolites

The important metabolites during *Drosophila* embryogenesis were identified based on nine authentic standards that include trehalose, glucose, proline, aspartic acid, glutamic acid, glycine, succinic acid, citric acid (or isocitric acid) and uric acid at a concentration of 0.1 mg/mL. The standards were co-injected during sample analysis. Two blank solutions were prepared by adding only extraction solvent and distilled water, respectively. No authentic standards were detected in either of the blank samples.

## Results and Discussion

### The metabolites change dramatically from early to late stage of *Drosophila* embryogenesis

**Figure 1:**
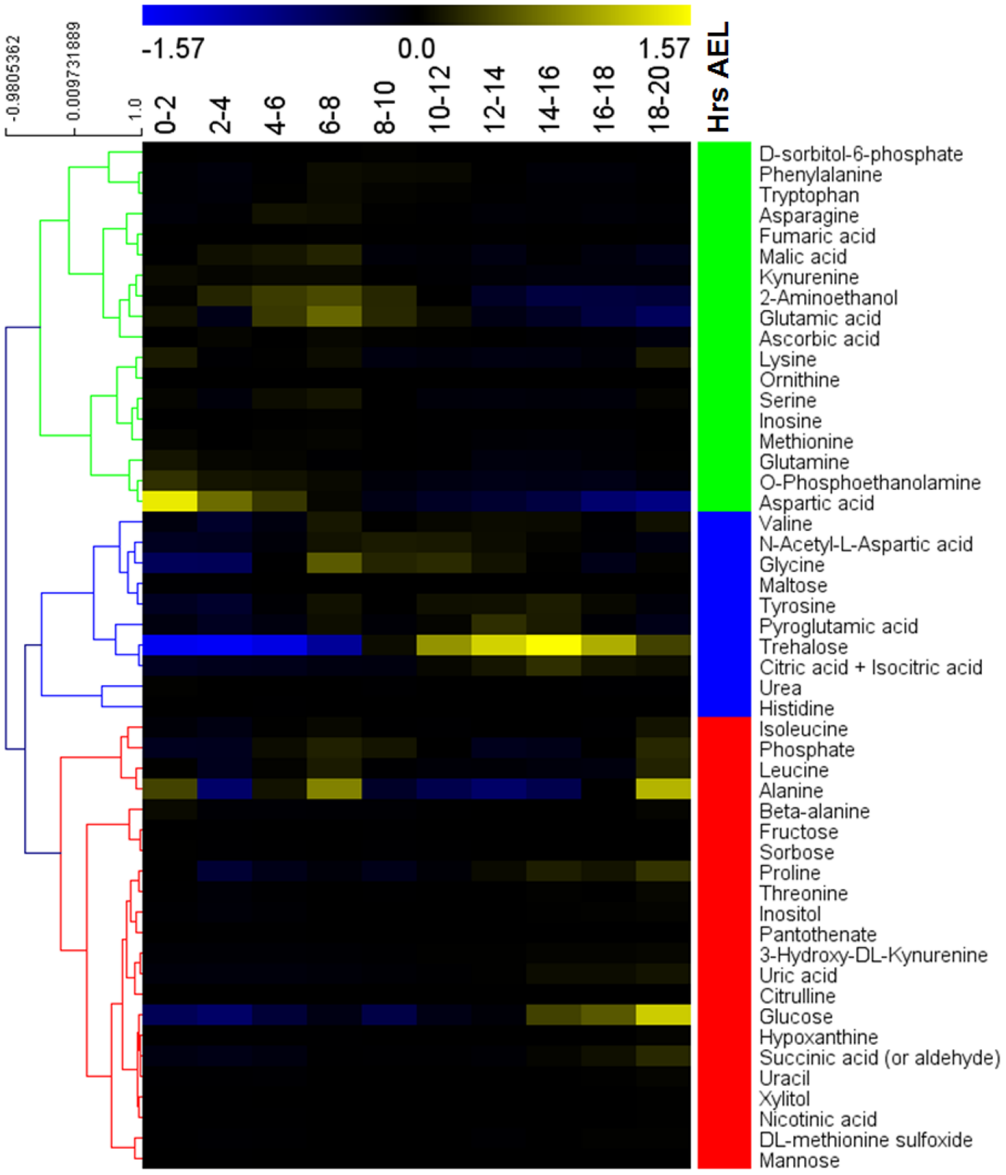
Heat map showing the widespread changes in metabolites during *Drosophila* embryogenesis. Metabolite levels were expressed relative to the average value of that metabolite throughout the development of embryo; the ratios were plotted on a color scale (top). Metabolites were discriminated hierarchically into three clusters which are represent for early (green), middle (blue) and late stage (red) of embryogenesis.

Since there was no previous study on the metabolomics of *Drosophila melanogaster* embryo, we decided to employ non-targeted GC/MS-based metabolic profiling to provide an instantaneous snapshot of the physiology of *Drosophila* during embryogenesis. After peak detection, fifty metabolites were tentatively identified by comparing the GC-MS data with the NIST and our in-house libraries (Table S1) to organize Metabolome data matrix (Table S2). These metabolites related to the central metabolic pathway including the metabolism of amino acid, sugar and nucleic acid, TCA cycle (Tricarboxylic acid cycle) and urea cycle. Then, hierarchical clustering analysis was performed to classify metabolites into clusters of different expression trends during embryogenesis. We found that these metabolites were discriminated into three clusters which represent the early, middle and late stage of embryogenesis (Figure 1). These results indicate that the metabolites change dramatically from early to late stage of *Drosophila* embryogenesis.

### The dynamic changes in the different developmental stages of *Drosophila* embryo are reflected in the metabolic profiling

**Figure 2:**
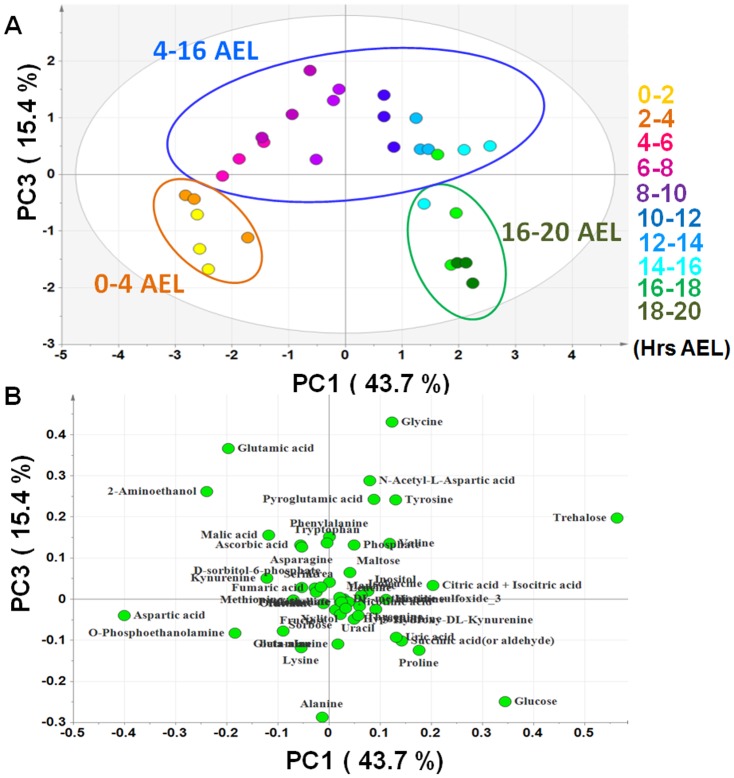
There is a high coincidence between the composition of metabolome and actual developmental stages of *Drosophila* embryo. Embryos were collected at 10 time points namely 0–2, 2–4, 4–6, 6–8, 8–10, 10–12, 12–14, 14–16, 16–18, 18–20 hrs AEL. All samples were analyzed in triplicates (n = 3). (**A**) PCA score plot shows 3 main clusters namely 0–4 hrs, 4–16 hrs and 16–20 hrs AEL, which is in total agreement with the developmental stages of *Drosophila* embryo (**B**) The loading plot shows the contributions of each metabolite on the discrimination in score plot according to the distance to the origin.

In order to clearly elucidate the correlation between the metabolome and actual developmental stages of *Drosophila* embryo, the metabolome data matrix was subjected to PCA. The results showed that in the principal component (PC) space formed by PC1 (43.7%) and PC3 (15.4%), the sample data separated into three distinct clusters namely “0–4 hrs AEL”, “4–16 hrs AEL” and “16–20 hrs AEL”. Specifically, PC1 clearly separates the different developing stages of the embryo. PC3 seems to contribute to the separation of the middle stage “4–16 hrs AEL” from the other stages (“0–4 hrs AEL” and “16–20 hrs AEL”) (Figure 2A). This grouping tendency was in complete agreement with the actual developmental processes occurring during *Drosophila* embryogenesis. Within the first 3 hrs AEL, the important phenomena include synchronized nuclear divisions, formation of the primary germ cells, pole cells and conversion of syncytium into cellular blastoderm instar larva [3], [29]. The gastrulation starts from 3 hrs AEL and lasts until 16 hrs AEL. During gastrulation, the single-layered blastula is reorganized into the gastrula composed of ectoderm, mesoderm, and endoderm. Then, it undergoes organogenesis, segmentation and the segregation of the imaginal discs. During the last stage (16–20 hrs AEL), the ventral cord continues retracting to complete embryogenesis [3], [29].

For the score plot interpretation, we focused on the distribution of metabolites in the loading plot based on their distance to the origin (Figure 2B). Several key metabolites were found to be important for the discrimination of the different developmental stages. Specifically, trehalose and glucose had positive contribution, while aspartic acid had negative contribution to the separation on PC1. On the other hand, glycine level is high and alanine level is low in gastrulation stage. These two metabolites were inversely correlated, which made the gastrulation stage separate from the early and late stages of embryogenesis according to PC3. Since these metabolites, related to sugar and amino acid metabolisms, are very important energy sources of the cell, energy metabolism may play an important role during the development of *Drosophila* embryo.

Taken together, we conclude that the dynamic changes in *Drosophila* embryogenesis can be explained by the changes in the composition of metabolome during different developmental stages of the embryo.

### A prediction model of *Drosophila* embryogenesis based on metabolome data was successfully developed

Since the metabolome was found to be correlated with biological activities during *Drosophila* embryogenesis, we speculated that it is possible to construct a prediction model based on metabolite information. A similar method was applied previously in zebrafish [30]. In this method, PLS, a regression extension of PCA, was utilized to find the relationship between two variables namely, metabolites and hours after egg laying (hrs AEL).

**Figure 3:**
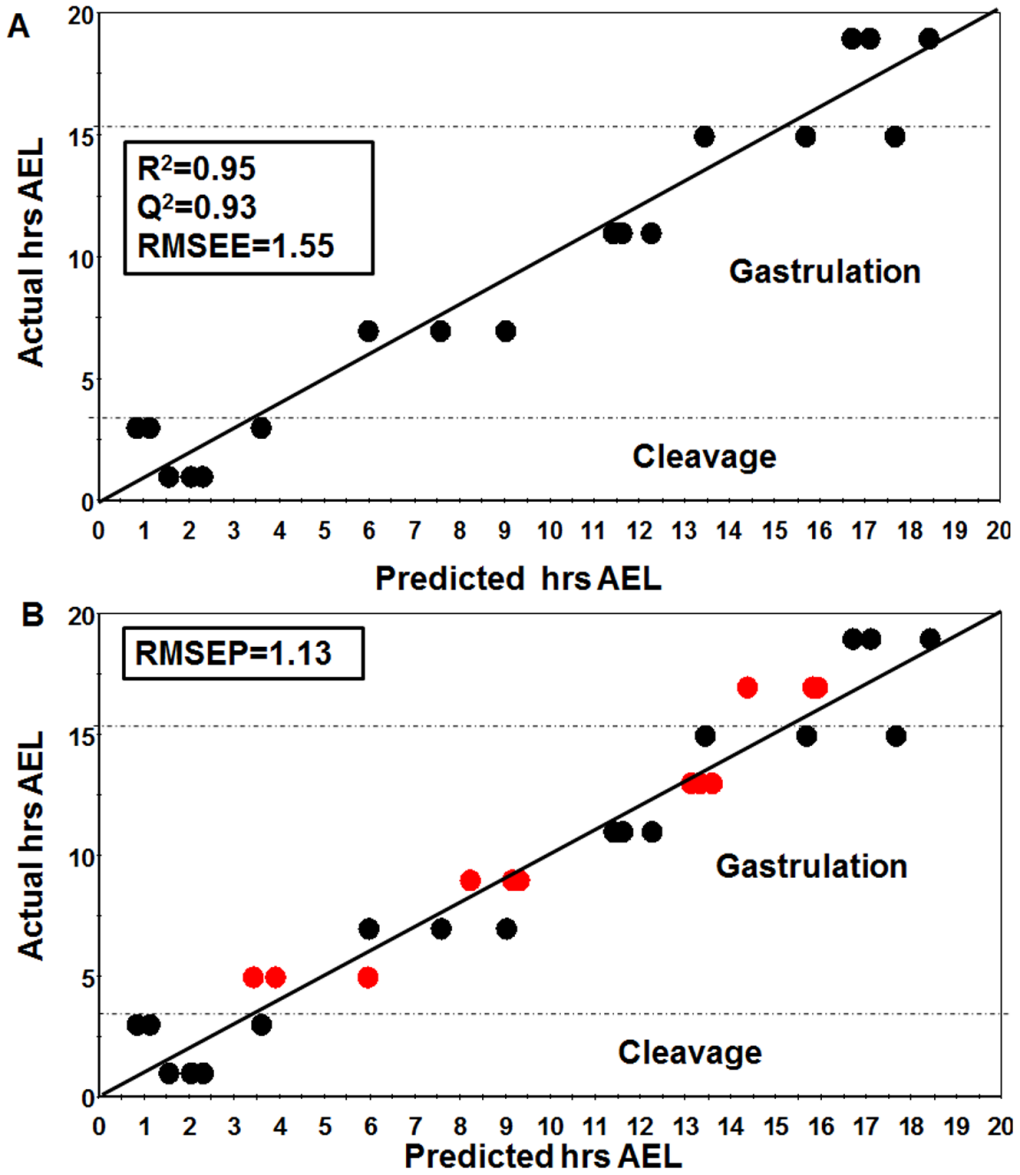
Partial Least Square was performed to construct a robust and accurate prediction model for *Drosophila* embryo developmental stages based on the metabolic data. Among 10 time points investigated, 4 time points including 4–6, 8–10, 12–14 and 16–18 hrs AEL were selected as the test set while the rest were used as training set. PLS regression was first performed with the training set by importing all compounds to the X-matrix while the actual AEL were imported to the Y-matrix. (**A**) A good correlation between metabolome data and developmental stages could be achieved. R^2^ and Q^2^>0.9 indicated an excellent predictive model. (**B**) Cross-validation of the model using the test set was fitted onto the prediction model constructed using the training set. The root mean square error was calculated to point out how well the observed hrs AEL matched with the real hrs AEL. RMSEP was not so different from RMSEE, showing that the model was validated.

Among the 10 time points investigated, 4 time points namely 4–6, 8–10, 12–14 and 16–18 hrs AEL were selected as the test set while the rest were used as the training set. PLS regression was first performed with the training set by importing the information of all 50 metabolites to the X-matrix while the actual AEL were imported to the Y-matrix. We found a good correlation between the metabolite information and developmental stages of *Drosophila* embryogenesis with goodness-of-fit (R^2^) and goodness-of-prediction (Q^2^) values of 0.95 and 0.93, respectively. The high R^2^ and Q^2^ values indicated an excellent predictive model (Fig. 3A). In order to verify our results, we added the test set into the model wherein they fit perfectly in the predicted regression line (Fig. 3B). Moreover, the root mean square error was calculated to determine how well the observed hrs AEL matched the actual hrs AEL. Result showed that the root mean square error of prediction (RMSEP = 1.13) was not significantly different from the root mean square error of estimation (RMSEE = 1.55), thus indicating that the regression model was valid. In conclusion, we successfully developed a prediction model of *Drosophila* embryogenesis based on metabolome data.

### Different metabolites play distinct roles during *Drosophila* embryogenesis

**Figure 4:**
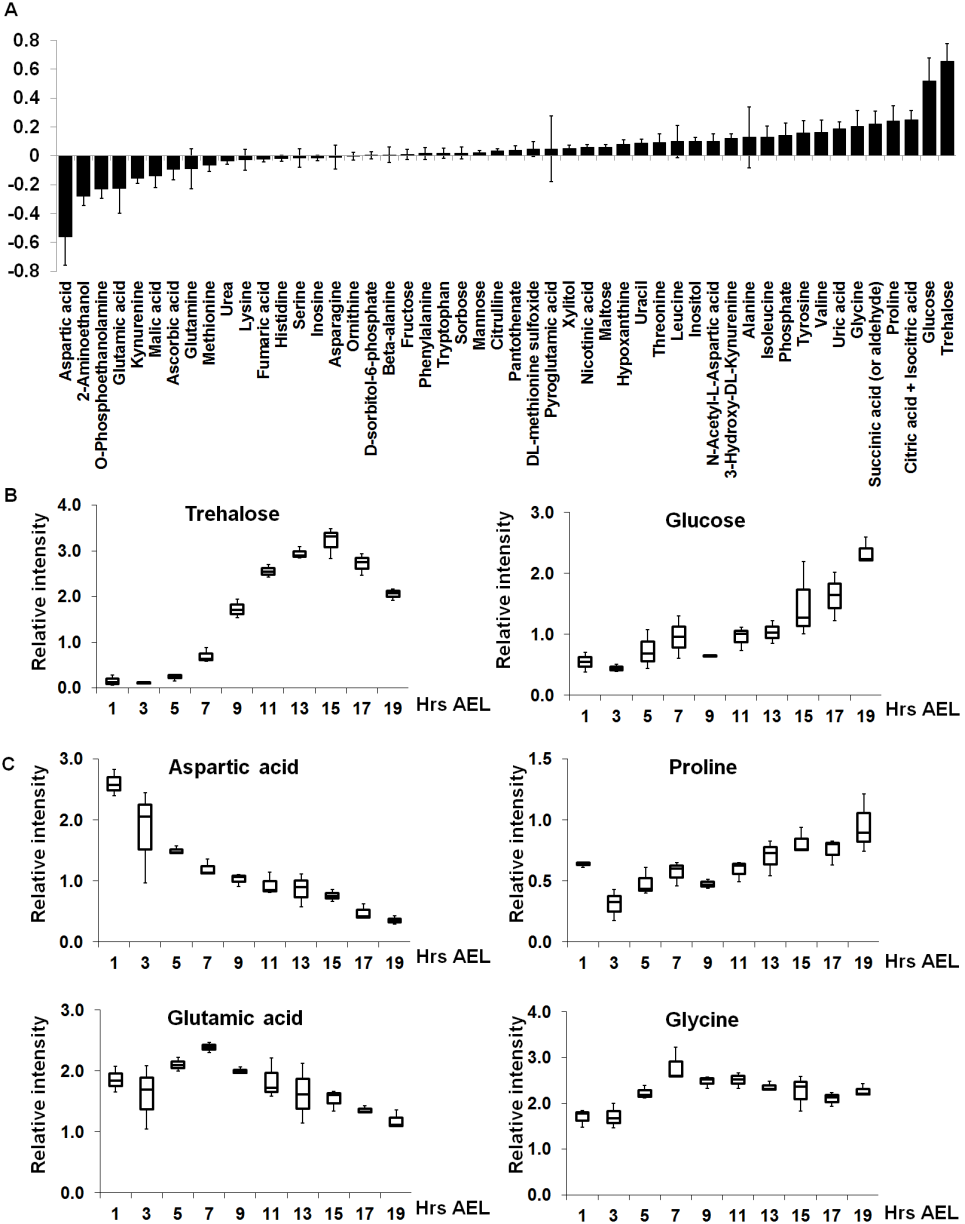
Each metabolite has distinct contributions to the development of *Drosophila* embryos. (**A**) The regression coefficient plot shows the correlation trend of each metabolite. The negative correlation indicated the important role during early embryogenesis and vice versa. The relative intensity of metabolites that are important in sugar and amino acid metabolism are shown in (**B**) and (**C**), respectively. The peak intensity of each compound was normalized based on ribitol internal standard.

The VIP (Variable Importance in the Projection) score of a predictor indicates the contribution of that variable to the model [30]. Since the average of squared VIP scores equals 1, the “greater than one rule” is generally used as a criterion for variable selection. Among the 50 metabolites detected, we found 11 metabolites that are important in our prediction model (Table 1). The identification of metabolites including trehalose, glucose, proline, aspartic acid, glutamic acid, glycine, succinic acid, citric acid (or isocitric acid) and uric acid were further confirmed by co-injecting standard compounds during sample analysis. On the other hand, the regression coefficient plot was utilized to see the correlation trend (negative or positive) of each metabolite to this model (Fig. 4A). Within this model, the metabolite which had a negative correlation was deduced as important during early embryogenesis and vice versa.

**Table 1:** Important metabolites for the development of *Drosophila* embryo.

Metabolite	VIP score
Trehalose	3.55
Aspartic acid	3.05
Glucose	2.80
2-Aminoethanol	1.51
Citric acid + Isocitric acid	1.35
Proline	1.31
*O*-Phosphoethanolamine	1.25
Glutamic acid	1.23
Succinic acid (or aldehyde)	1.20
Glycine	1.11
Uric acid	1.01

Among the metabolites related to sugar metabolism, we found that trehalose and glucose played important roles during the development of the embryo. Although both sugars were increased over time, we found that trehalose, which had highest VIP score (Table 1), accumulated in an abundant level during gastrulation (Fig. 4A, B). Previous studies reported that trehalose is present as an energy source in the *Drosophila* haemolymph as early as the larval stage [31], [32]. In addition, expression data have also shown that two *Drosophila* trehalose transporters, encoded by the *Tret 1-1* and the *Tret 1-2* genes, are highly expressed during gastrulation while the *Treh* gene, encoding for the enzyme that converts trehalose into glucose, is expressed throughout embryogenesis [5]. Therefore, we propose that from 8 to 16 hrs AEL trehalose is synthesized and transferred to the tissues that require it as a carbon source. Although in larval stage glucose in the fat body is utilized to generate trehalose [31], [32], trehalose used in embryogenesis must be generated from other sources, since the level of glucose is quite low in the first 14 hrs AEL (Fig. 4B). Afterwards, the level of trehalose decreases, while the level of glucose increases from 16 to 20 hrs AEL (Fig. 4B). Taking account of these observations, we suggest that trehalose is used as the energy source for glycolysis to supply glucose for the cells during late stage of embryogenesis.

Neurogenesis of *Drosophila* embryo starts from 3 hrs AEL and lasts until the end of embryogenesis. During neurogenesis, the differentiation of central and peripheral nervous system together with head involution occur from 9 to 13 hrs AEF, while the retraction of ventral cord takes place from 16 to 20 hrs AEL. Moreover, previous study also reported that trehalose transporter 1 involves in trehalose import into peripheral tissues to regulate the level of trehalose in insect [33]. Hence, there is a strong correlation between the increase of trehalose level and neurogenesis in *Drosophila* embryo. Altogether, we suggest that trehalose and glucose are the main carbohydrate source to supply energy and an appropriate balance in trehalose-glucose ratio is important for the development of *Drosophila* embryo.

From this study, we also deduced that amino acid metabolism is essential during *Drosophila* embryogenesis and different amino acids appear to play distinct roles in different developmental stages of the embryo. In our model, the amino acids with high VIP scores (aspartic acid, glutamic acid, glycine and proline) (Figure 4C) belonged to the glucogenic amino acid group, which could be converted into glucose via gluconeogenesis [34]. Based on the detected level of each metabolite during embryogenesis, it should also be noted that aspartic acid is only important for the cleavage stage (0–4 hrs AEL) while both glutamic acid and glycine are critical for early gastrulation (6–8 hrs AEL). In fact, insects do not carry out gluconeogenesis from lipid substrates because the glyoxylate cycle is either totally or partially inoperative [35]. Since *Drosophila* egg is a closed system and zygotic transcription is not required until interphase of the 10^th^ nuclear cycle [36], the embryo must be endowed with an abundance of maternally-supplied products and these amino acids possibly provide another pathway to control energy production during embryogenesis. In addition, very similar results, especially on the metabolites related to amino acid metabolisms have been recently reported by Tennessen *et al.* [37], supporting the hypothesis that amino acids play an important role in maintaining the energy during the development of *Drosophila* embryo.

On the other hand, aspartic acid had the highest contribution to the separation of the early stage (0–4 hrs AEL) from the other periods (6–20 hrs AEL) (Fig. 4A, C). In *Drosophila* embryo, the nuclear division cycle consists of S and M phases without any intervening gap phases like G1 or G2 phase. Thus, the initial division cycles proceed rapidly, ranging from 10 to 25 minutes, as compared to the typical cell cycle duration of 24 hours [4]. Our observations indicate that aspartic acid, which is related to purine and pyrimidine synthesis [38], might be a crucial element for supplying substrates for DNA replication during the rapid nuclear division cycles of early *Drosophila* embryogenesis.

By using GC/MS, 2-aminoethanol and *O*-Phosphoethanolamine were also tentatively detected. 2-aminoethanol is the second-most-abundant head group for phospholipids [39] while *O*-Phosphoethanolamine is a precursor of phospholipid synthesis and a product of phospholipid breakdown [40].

In summary, our study indicated that different metabolites play distinct roles during the development of the *Drosophila* embryo, which may reflect the biological changes in the cell. Based on the level of metabolites observed, we were also able to extrapolate their implications in the various pathways that may contribute to the overall development of the *Drosophila* embryo. However, we cannot exclude the possibility that our analysis provides a snapshot of the metabolic rate of *Drosophila* embryo and therefore, depending on the developmental stage, the metabolic profile could be different in various cell types or tissues.

## Conclusion

Comparing to the huge database on genomics, transcriptomics and proteomics related to developmental biology, information from metabolomics studies are limited to just a few model organisms such as yeast, zebra fish or frog [41]–[47]. To our knowledge, this is the first report of a precision multivariate model which can be used to predict one developmental stage of *Drosophila* embryo based on metabolome data. The present study has shown that the distinct metabolic profiling coincided with the actual separation based on developmental stages of *Drosophila* embryo.

It has been proposed that prior to gastrulation, amino acids are consumed as the primary source of energy for early frog embryogenesis [43]. On the other hand, studies on zebra fish embryogenesis found that some metabolites related to glycolysis and TCA cycle served fundamental roles in a developing embryo [44]. These studies suggest that energy metabolism may play an essential role during embryogenesis. Our work complements this hypothesis by proposing that sugar and amino acid metabolisms are important energy sources during *Drosophila* embryogenesis.

Finally, this study offers a general view of the metabolic pathways that are active during *Drosophila* embryogenesis. Furthermore, it can serve as a basis for further investigation of the mechanism of embryogenesis in *Drosophila* as well as other developmental studies.

## Supporting Information

Table S1 **Metabolic profile during** ***Drosophila melanogaster*** **embryogenesis.**
(DOCX)

Table S2 **Metabome analysis of** ***Drosophila melanogaster*** **during embryogenesis (Normalized data).**
(XLSX)
