# Supplementary material for: Metabolome Analysis of Drosophila melanogaster during Embryogenesis
Source: PLoS One. 2014 Aug 14;9(8):e99519. doi: 10.1371/journal.pone.0099519 (PMC4133167; doi:10.1371/journal.pone.0099519)
Supplement: Table S1 — Metabolic profile during Drosophila melanogaster embryogenesis. (DOCX) [file pone.0099519.s001.docx]

Table S1: Metabolic profile during *Drosophila melanogaster* embryogenesis.

| **Compound Name** | **Retention time (min)** | **Compound Name** | **Retention time (min)** | **Compound Name** | **Retention time (min)** |
| --- | --- | --- | --- | --- | --- |
| Alanine | 5.32 | Aspartic acid | 9.38 | Mannose | 12.10 |
| Valine | 6.56 | Methionine | 9.43 | Glucose | 12.18 |
| Urea | 6.82 | Pyroglutamic acid | 9.48 | Lysine | 12.41 |
| 2-Aminoethanol | 7.09 | Glutamic acid | 10.20 | Histidine | 12.49 |
| Phosphate | 7.10 | Phenylalanine | 10.33 | Tyrosine | 12.56 |
| Leucine | 7.12 | N-Acetyl-L-Aspartic acid | 10.52 | Ascorbic acid | 12.58 |
| Isoleucine | 7.34 | Asparagine | 10.60 | Pantothenate | 12.89 |
| Proline | 7.43 | Xylitol | 10.79 | Inositol | 13.49 |
| Nicotinic acid | 7.46 | Glutamine | 11.38 | Uric acid | 13.56 |
| Glycine | 7.49 | *O-*Phosphoethanolamine | 11.44 | Kynurenine | 14.18 |
| Succinic acid(or aldehyde) | 7.56 | Citric acid + Isocitric acid | 11.66 | Tryptophan | 14.35 |
| Uracil | 7.80 | Ornithine | 11.69 | D-sorbitol-6-phosphate | 14.80 |
| Fumaric acid | 7.90 | Hypoxanthine | 11.71 | 3-Hydroxy-DL-Kynurenine | 15.48 |
| Serine | 7.95 | Citrulline | 11.74 | Inosine | 16.25 |
| Threonine | 8.19 | Sorbose | 12.00 | Maltose | 16.91 |
| Beta-alanine | 8.62 | DL-methionine sulfoxide | 12.01 | Trehalose | 17.02 |
| Malic acid | 9.10 | Fructose | 12.06 |  |  |
